# DeepEmSat: Deep Emulation for Satellite Data Mining

**DOI:** 10.3389/fdata.2019.00042

**Published:** 2019-12-10

**Authors:** Kate Duffy, Thomas Vandal, Shuang Li, Sangram Ganguly, Ramakrishna Nemani, Auroop R. Ganguly

**Affiliations:** ^1^Sustainability and Data Sciences Laboratory, Department of Civil and Environmental Engineering, Northeastern University, Boston, MA, United States; ^2^Ames Research Center, NASA, Mountain View, CA, United States; ^3^Bay Area Environmental Research Institute, Petaluma, CA, United States

**Keywords:** remote sensing, machine learning, deep learning, atmospheric correction, emulator

## Abstract

The growing volume of Earth science data available from climate simulations and satellite remote sensing offers unprecedented opportunity for scientific insight, while also presenting computational challenges. One potential area of impact is atmospheric correction, where physics-based numerical models retrieve surface reflectance information from top of atmosphere observations, and are too computationally intensive to be run in real time. Machine learning methods have demonstrated potential as fast statistical models for expensive simulations and for extracting credible insights from complex datasets. Here, we develop DeepEmSat: a deep learning emulator approach for atmospheric correction, and offer comparison against physics-based models to support the hypothesis that deep learning can make a contribution to the efficient processing of satellite images.

## 1. Introduction

Contemporary satellite remote sensing is responsible for contributing Earth science data to public repositories at an unprecedented volume (Overpeck et al., 2011). This abundant data has drawn interest to applying machine learning (ML) for data mining (Castelluccio et al., 2015; Xie et al., 2016; Mou et al., 2017), climate data downscaling (Vandal et al., 2017), and to advance process understanding in Earth sciences (Reichstein et al., 2019). These emerging success stories suggest that machine learning has potential for extracting credible insights from complex datasets in multiple domains.

Land surface products such as crop forecasts, vegetation indices, snow cover, and burned area are derived from a basic parameter termed surface reflectance (SR). SR is a characteristic of the Earth's surface and is produced from raw, top of atmosphere (TOA) observations by removing the effects of atmospheric scattering and absorption. This process, termed atmospheric correction (AC) allows greater comparability between observations across space and time. However, physically based numerical models for atmospheric correction are too computationally intensive to be calculated in real time, relying instead on look-up tables with precomputed values. Additionally, atmospheric correction models must be tuned for new sensors, which may have short operational lifespans.

Here, we examine the hypothesis that deep learning can make contributions to the efficient processing of satellite data. We develop an experiment in atmospheric correction and present results to suggest that a deep learning model can be trained to emulate a complex physical process. Results are presented to demonstrate the emulator's stable retrieval of surface reflectance when validated against traditional physics-based models.

## 2. Materials and Methods

### 2.1. Related Work

#### 2.1.1. Atmospheric Correction

**Figure 1:**
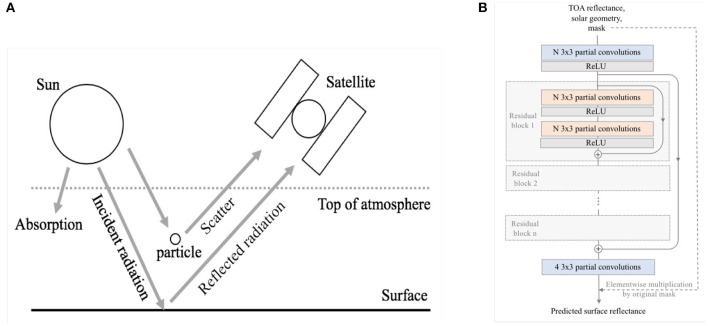
**(A)** Physics-based atmospheric correction algorithms simulate reflection and scatting processes at the Earth's surface and in the atmosphere. **(B)** Architecture of the emulator model, a modified ResNet with n residual blocks and N hidden units.

Figure 1 provides a schematic drawing of radiative transfer processes in the atmosphere. Non-learning approaches to AC use physical modeling and empirical relationships to retrieve surface reflectance from observations contaminated by atmospheric scattering and absorption processes that occur in the paths between the sun, the Earth's surface, and the satellite sensor.

The algorithm used to derive MOD09GA, the daily SR product from NASA's Moderate Resolution Imaging Spectroradiometer (MODIS) corrects for gases, aerosols and Raleigh scattering. Due to prohibitive computational complexity, MOD09GA relies on look-up tables for aerosol retrieval and for precomputed SR retrieved according to atmospheric conditions (Vermote and Kotchenova, 2008). MAIAC is a newer algorithm that uses time series and spatial analysis to detect clouds, retrieve aerosol thickness and retrieve SR (Lyapustin et al., 2011a,b, 2012). MAIAC uses two algorithms, depending on whether the observation area is stable or undergoing rapid change, as classified by the change detection algorithm (Lyapustin et al., 2012). These approaches, and other state-of the art approaches including Fast Line-of-sight Atmospheric Analysis of Spectral Hypercubes (FLAASH) rely on sensor calibration and retrieval of accurate atmospheric conditions (Cooley et al., 2002).

#### 2.1.2. Machine Learning in Remote Sensing

ML techniques have been applied to remote sensing with results that enhance upon non-learning methods. ML has been used to implement empirical bias corrections to MODIS measurements (Lary et al., 2009). In atmospheric correction, a Support Vector Machine (SVM) has been used to predict SR from TOA reflectance with good agreement between reflectance products retrieved from the ML method and from two radiative transfer models (Zhu et al., 2018). This approach trains a separate model for each band.

Prior work also has blended data produced by multiple satellites to obtain synthetic images with enhanced spatial or temporal resolution (Gao et al., 2006). Convolutional Neural Networks (CNN) have been used in remote sensing for tasks such as land cover classification, object detection and precipitation downscaling, which make use of local correlation structures (Castelluccio et al., 2015; Long et al., 2017; Mou et al., 2017; Vandal et al., 2017).

Outside of the remote sensing domain, CNNs have been used for style transfer, where image content is preserved and image texture is modified (Gatys et al., 2016). This problem has similarities to the problem of atmospheric correction, in which we wish to preserve semantic structure of the image while applying some effect. In atmospheric correction, this includes reversing the blue shift and reducing the blurring caused by passage through the atmosphere.

#### 2.1.3. Deep Residual Networks

Deep CNNs can reach an accuracy saturation, where increasing depth is associated with decreasing training accuracy (He et al., 2015). It is understood that a stack of nonlinear layers has a difficult time learning an identity mapping, thus a difficult time preserving the resolution of images. He et al. introduced deep residual nets (ResNets) in 2015. ResNets outperform state of the art methods in several image recognition competitions and are believed to be generalizable to vision and non-vision tasks.

### 2.2. Datasets

Data from two satellites are used in this experiment: Terra and Himawari-8. Terra is low earth orbit (LEO) satellite carrying the MODIS sensor. Terra travels in a north-south direction, passing over the poles and crossing the equator at a near- orthogonal angle. As the Earth rotates, the satellite scans the Earth's surface over a span of hours to days. The Japan Meteorological Agency geostationary (GEO) satellite Himawari-8 carries the Advanced Himawari Imager (AHI) sensor, which has similar spectral characteristics to MODIS. In contrast to LEO satellites, GEO satellites orbit in the same direction as the Earth's rotation, staying “stationary” when viewed from the Earth's surface. GEO satellites orbit at a higher altitude than LEO satellites, but have the capacity to observe locations within their view with sub-hourly frequency.

The Advanced Himawari Imager TOA reflectance described below comprises the input to the emulator model. Surface reflectance produced from Terra's MODIS is the target for prediction. An Advanced Himawari Imager SR product, also calibrated against MODIS SR, provides performance comparison with a physically-based model.

#### 2.2.1. AHI TOA Reflectance

To prepare TOA reflectance, raw scans from Himawari-8 are georeferenced and assembled into a gridded format. Pixel values are converted to TOA reflectance according to the Himawari-8/9 Himawari Standard Data User's Guide, Version 1.2 (Japan Meteorological Agency, 2015). The resulting full disk TOA is reprojected into geographic (latitude-longitude) projection with a 120° by 120° extent and 0.01° resolution. The domain, extending from 85°E to 155°W and 60°N to 60°S, is divided into 6° by 6° tiles. Full disk observations are repeated every 10 min. This gridded product (HM08_AHI05) is publicly available (https://www.geonex.org/). Four bands—blue, green, red, and near infrared (NIR)—are selected from AHI TOA data (Table 1). The data is treated as a multi-channel image, concatenated with two additional channels of solar zenith and solar azimuth angles.

**Table 1:** Summary of target SR bands: MODIS Terra Level 1B and AHI-12 SR product.

	**Center wavelength (nm)**	
**Band**	**MODIS Terra**	**AHI**
Blue	470	471
Green	555	510
Red	648	639
NIR	858	857

*Center wavelengths are shifted slightly due to the different sensors*.

#### 2.2.2. MODIS Terra Surface Reflectance

MOD09GA is a seven-band surface reflectance product computed from Terra MODIS sensor (Vermote and Kotchenova, 2008). This MODIS SR product, which is validated with ground observations, provides a standard for the calibration of other atmospheric correction algorithms (Liang et al., 2002). Four bands from MOD09GA, corresponding to four AHI bands, are selected based on available spatial resolution (Table 1). MOD09GA is resampled from the distributed 1km x 1km sinusoidal projection to a 0.01° geographic projection, described above, using GIS tools.

#### 2.2.3. AHI Surface Reflectance

GEO surface reflectance is retrieved from AHI TOA reflectance using the MAIAC algorithm (henceforth referred to as MAIAC SR). MAIAC is a semi-empirical algorithm originally developed for MODIS and adapted to perform SR retrievals for Himwari-8 AHI. Performance of MAIAC is evaluated by comparison with MOD09GA. The projection and resolution are identical to AHI TOA reflectance, described above. This product (HM08_AHI12) is released as a provisional product and is available upon request (https://www.geonex.org/).

All data belongs to a 3 month period of December 2016 through February 2017. We use observations over the Australian continent. This landmass is chosen as it provides a large contiguous landmass with a variety of land cover classes with which to train a flexible emulator. Where satellite images are affected by missing pixels due to clouds, aerosols, and water bodies, we select images for training and testing only if they contain 80% valid pixels or greater. We create and apply a composite mask to standardize valid pixels between all images from the same date. Furthermore, all reflectances are normalized to intensity between 0 and 1.

### 2.3. Proposed Method

In this section we introduce a residual neural network to predict MODIS-like multispectral SR from TOA reflectance and solar geometry. This emulator model is trained with MODIS SR as the target, with the objective of emulating the MOD09GA atmospheric correction algorithm.

#### 2.3.1. Network Architecture

We modify ResNets with long and short skip connections, as defined by He et al. and as depicted in Figure 1 (He et al., 2015). In this modified architecture, patch dimensions (width and height) are preserved throughout the network, as only relatively local information is necessary to retrieve pixelwise SR. Input patches are treated as six channel images, with four wavelength bands and two solar angle bands. Output images are four channel images with four wavelength bands.

ResNets and CNNs with varying numbers of residual blocks and hidden units are trained to determine the optimal architecture for this application. Models with partial convolutions (ResNet-P and CNN-P) and without partial convolutions (ResNet and CNN) are tested.

#### 2.3.2. Partial Convolutions

Missing pixel values pose a processing problem in CNNs. When they fall within the convolutional window centered around a neighboring pixel, missing values create anomalous output, or edge effects. Partial convolutions offer a semantically aware method for normalization of output values that performs well on irregularly shaped holes. In this method, a binary mask is used to calculate scaling factors that adjust outputs according to the number of valid inputs.

Given **M**, a binary mask denoting the positions of valid and invalid pixels, *x*, the values in the sliding convolution window, and **W**, the convolution window weights, the output of the partial convolution layer is defined as:

(1) $$\begin{array}{l} x^{\prime }=\{\begin{array}{ll} W^{T}(x\;\cdot \;M)\frac{1}{sum(M)}+b & \text{if sum}(\text{M})>\text{0}\\ 0 & \text{if sum(M) = 0} \end{array} \end{array}$$

Convolutions with some or all valid pixels in the window are properly weighted and accepted as a valid response; convolutions with no valid pixels are not accepted. In each step, the binary mask is updated where a valid response was made, progressively shrinking holes. We adapt partial convolutions to prevent ill effects in processing satellite images with missing pixels due to clouds, cloud shadows and water bodies. Partial convolutions also have the desirable effect of eliminating edge effects in patches when used in combination with zero padding.

Partial convolutions are implemented in TensorFlow using the convolutional operation described by Liu et al. (2018). While partial convolutions shrink holes in images, our approach reapplies the original mask to the model output. This preserves the interpretability of the results, as ground truth surface reflectance values are not available for all missing pixels that are inferred through inpainting. We compare the results of both models with partial and regular convolutions.

#### 2.3.3. Implementation Details

##### 2.3.3.1. Loss Function

A mean square error loss function with weight regularization is employed to learn the regression based convolutional neural network written as

(2) $$\begin{array}{l} ℒ(\Theta )=\frac{1}{N}\sum_{i=1}^{N}{\left(y-f(x|\Theta )\right)}^{2}+\lambda {||\Theta ||}_{2} \end{array}$$

where Θ consists of weights and bias parameters of neural network *f*.

##### 2.3.3.2. Experimental Setup

Each network is trained on 50 by 50 pixel image patches randomly extracted using Adam optimization with β_1_ = 0.999, β_2_ = 0.9, ϵ = 1*e* − 8, a batch size of 30, and learning rate of 0.001 (Kingma and Ba, 2014). Observations covering southern Australia are used for training with northern Australia set aside for testing. By geographically dividing training and testing data, we ensure that testing images are covering a region totally unseen in the training examples. The model, implemented using TensorFlow, is trained for 300,000 iterations on one NVIDIA GeForce GTX 1080ti graphics card over approximately 7 h.

#### 2.3.4. Implementation Details

The reflectance product generated by the emulator is validated by comparison with MODIS SR (MOD09GA). Reference to a comprehensively validated SR product is a standard assessment for new SR products (Feng et al., 2012). In addition to direct comparison with MOD09GA, performance of emulator SR retrieval is benchmarked by comparison with the MAIAC SR product, also generated from Himawari-8 TOA reflectance. This MAIAC algorithm has been calibrated using agreement with MODIS SR, and provides a comparison between the emulator and a physically based model using the same sensor.

We use root mean square error (RMSE) as a metric of distance between prediction and MODIS SR, and evaluate each spectral band individually. RMSE is computed on the dimensionless pixel reflectance, which takes values between 0 and 1. To further assess the goodness of fit, Pearson's r, and the related metric *R*^2^, are statistical measures calculated to determine the amount of variation of data explained by the model. *R*^2^ always falls between 0 and 1, with a higher *R*^2^ value indicating better fit of the model to the data. Pearson's r and *R*^2^ are common metrics in the remote sensing domain to measure the coherence between images for validation purposes (Vinukollu et al., 2011; Tang et al., 2014). Additionally, we compute mutual information (MI) as an image matching metric. Mutual information is a dimensionless quantity that expresses how much information one random variable gives us about another. MI here is calculated with respect to the MODIS SR product.

## 3. Results

### 3.1. Compared Methods and Models

For the prediction of surface reflectance, we compare plain CNNs and ResNets of varying depth and width. We test models grid-search style with 1–5 residual blocks and 16–128 hidden units. For modified ResNet, the 5 residual block architecture with 64 hidden units per layer achieves the best performance. For CNN without residual connections, a 4 layer architecture with 64 hidden units per layer performs best. We test each of these models with partial convolutions (referred as ResNet-P, CNN-P) and regular convolutions (referred as ResNet, CNN). As shown in Table 2, ResNet-P achieves the best performance among the four models, with 19% lower RMSE than that of CNN.

**Table 2A:** Performance metrics by band and for full spectrum for ResNet, ResNet with partial convolutions (Resnet-P), CNN, CNN with partial convolutions (CNN-P), and MAIAC.

**Model**	**Testing RMSE (10**^****−2****^**)**					*******R***^**2**^****				**Mutual information**
	**Blue**	**Green**	**Red**	**NIR**	**Full**	**Blue**	**Green**	**Red**	**NIR**	
ResNet-P	**0.80**	**1.4**	2.5	**2.8**	**1.9**	0.54	0.63	**0.86**	**0.83**	0.94
ResNet	1.0	1.7	**2.2**	2.9	2.2	0.46	0.51	0.82	0.81	0.92
CNN-P	1.0	1.9	2.4	3.0	2.2	0.54	0.56	0.82	0.78	0.92
CNN	1.1	1.9	2.4	3.2	2.3	**0.86**	**0.68**	0.70	0.68	0.88
MAIAC	1.1	2.1	3.2	5.6	3.6	0.39	0.50	0.85	0.77	**0.96**

*Best values are bolded*.

**Table 2B:** Performance metrics by band and for full spectrum for ResNet-P and MAIAC across three land cover types.

**Model**	**Land cover**	**Testing RMSE (10**^****−2****^**)**					**Mutual information**
		**Blue**	**Green**	**Red**	**NIR**	**Full**	
Emulator	Savanna	0.70	1.2	2.2	2.7	1.9	0.95
	Shrubland	0.80	1.1	2.3	2.3	1.8	0.95
	Forest	0.70	1.2	2.7	4.0	2.5	1.0
MAIAC	Savanna	1.1	1.7	1.9	3.7	2.4	0.97
	Shrubland	1.0	2.1	3.3	6.6	4.0	0.97
	Forest	1.0	1.5	1.3	5.2	2.9	0.98

We also evaluate the contribution of solar angle information to performance by training the model with and without solar angle information. We find that this additional information has a negligible impact on prediction accuracy.

### 3.2. Prediction of Surface Reflectance

**Figure 2:**
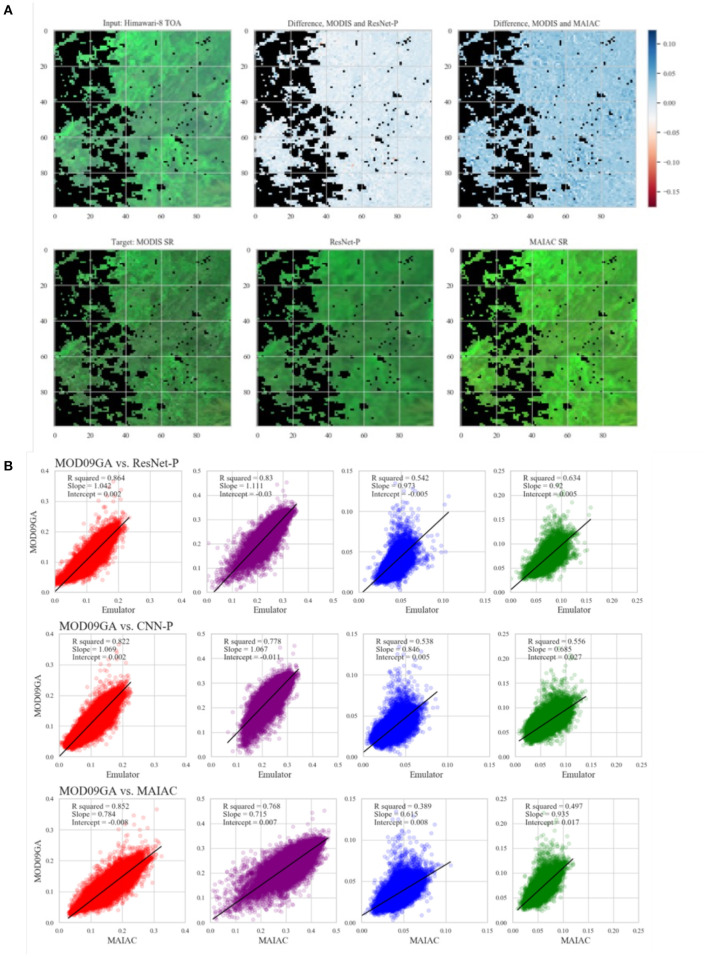
**(A)** Counterclockwise from top left: input TOA reflectance, target MODIS SR, emulator SR, MAIAC SR, visualization of difference between MODIS and two SR products. **(B)** Agreement between model predictions (ResNet-P, CNN-P, and MAIAC) and MODIS SR for 4 bands. Results are presented for one typical 600 × 600 pixel image from the testing set.

Performance of the emulator is evaluated by comparison with MODIS SR, and benchmarked by comparison of MAIAC SR to MODIS SR (Figure 2). We evaluate RMSE for each wavelength and also for the full spectrum in Table 2. This measure of distance suggests ResNet-P as the best performing model, with error on the order of 10% or less of normalized pixel values. Predictions for a representative testing set tile plotted are pixelwise against ground truth data from the MODIS product and presented in Figure 2. *R*^2^ and best fit (slope and intercept) by wavelength for the testing set are presented in Figure 2. The *R*^2^ values evidence high agreement between the emulator predictions and the MODIS retrievals of surface reflectance for the red and NIR bands, and lesser predictive power for the green and blue bands. Outliers are observed in all bands, particularly where MODIS reflectance exceeds the prediction by the other model. Outliers in SR are generally caused by localized light sources or reflections. Mutual information, which captures both linear and nonlinear dependence, also suggests ResNet-P as the best model among those compared.

In comparison with MAIAC SR, the emulator SR results in lower RMSE and better *R*^2^ agreement in all bands. MAIAC outperforms the emulator in MI score. This result suggests that some aspects of MODIS SR may be better captured by a deep learning model, while other aspects are better captured by the physical model.

### 3.3. Stability of Retrievals by Land Cover Type

The Australian continent is host to multiple land cover types, including savannas, shrublands, and forest, as delineated by the Collection 6 MODIS Land Cover Product (Sulla-Menashe and Friedl, 2018). We assess the stability of SR retrieval across land cover types by presenting performance metrics for the emulator across tiles dominated by these three homogeneous land cover types: savanna, shrubland, and forest.

Metrics for each land cover class are presented in Table 2. The results suggest good generizability of the emulator, with comparable performance across savanna and shrubland, and poorer performance for forested area, particularly driven by poorer performance for the NIR band.

### 3.4. Partial Convolutions for Missing Data

We evaluate the performance of partial vs. regular convolutions to handle missing pixels. We find that use of partial convolutions produces a 4% reduction in RMSE. Because partial and regular convolutions perform identically for regions of valid pixels, we would expect the differential in RMSE between the two techniques to be strongly correlated to the quality of the image, i.e., the number of missing pixels.

## 4. Discussion

Herein is presented DeepEmSat, an emulator for physically-based atmospheric correction. The objective of this work is to test the hypothesis that deep learning can make a contribution to the efficient processing of reflectance observations from Earth-observing satellites. The premise examines the possibility that a sufficiently complex neural network can learn the potentially nonlinear mapping of TOA reflectance to SR. This hypothesis also examines the possibility that semantic relationships between pixels in reflectance observations can be harnessed by convolutional networks.

The results of this study suggest that deep learning emulators can make some contribution to efficient processing of satellite images. The evaluation metrics comprise linear measures of similarity (Euclidean distance, linear correlation) as well as a measure from probability theory (mutual information). These metrics may describe different aspects of the relationship between variables. However, it is important to recall that physically-based AC algorithms contain biases and uncertainties of their own, making comparison with existing SR products an imperfect method of validation.

By training and testing on separate geographic regions, we demonstrate the generalizability of the model for locations outside of the training dataset. Our assessment of emulator performance over various land types suggests also stable SR retrieval by the emulator model. We demonstrate the improvement of model accuracy with the addition of partial convolutions, although more rigorous investigation of this effect is warranted. Through comparison with MAIAC, a physically based AC algorithm, we demonstrate the relatively strong performance of the emulator in generating MODIS-like surface reflectance from GEO TOA observations.

Diurnal, seasonal, and annual variation in solar angle limits comparability between reflectance observations from different times. Therefore, validation of emulator retrievals is limited to the approximate time of MODIS observations. Inferences for other locations, times of day, and seasons should be interpreted with caution. Our dataset is comprised only of observations over Australia. In future work, training data could be augmented with annual observations and also with MODIS Aqua satellite, which passes daily at 1:30 p.m. local time.

## 5. Conclusion

Prior studies have leveraged machine learning to extract insights from complex Earth science datasets. Here, we examine the hypothesis that a deep learning emulator of a physical model can contribute to efficient satellite data processing. In this work, domain knowledge from atmospheric science is used in covariate selection and design of model architecture. Our results suggest that further work, including development of principled approaches to the blending of physical and data science methods, will be useful to extract insights from a growing volume of remotely sensed Earth science data.

## Data Availability Statement

The datasets generated for this study are available on request to the corresponding author.

## Author Contributions

KD performed the experiment, analyzed the results, and led the preparation of the manuscript. TV assisted with technical aspects including implementation of the model and contributed to the problem definition and manuscript. SL prepared and provided the datasets that were used. SG, RN, and AG provided helpful guidance in conception of the project and design of the experiment.

### Conflict of Interest

The authors declare that the research was conducted in the absence of any commercial or financial relationships that could be construed as a potential conflict of interest.
